# Decision making in chronic subdural haematoma patient referrals in an octogenarian and nonagenarian population – a multi-centre perspective

**DOI:** 10.1007/s11845-026-04330-1

**Published:** 2026-05-14

**Authors:** Joshua MacArthur, Sayan Biswas, Lareyna McMenemy, Keneth Kodituwakku, Gokul Raj Krishna, Saskia Swannack, Samuel Wiltshire, Mohammad Saleemi, Helena Thompson, Anand Pandit, Ved Sarkar, Ahmed Toma, Emily Feilding, K. Joshi George

**Affiliations:** 1 Faculty of Biology, Medicine and Health, University of Manchester, Manchester, Greater Manchester UK; 2 Specialized Foundation Doctor Training Programme, Edge Hill University, Omskirk, England L39 4QP UK; 3 Department of Surgery, Manchester University NHS Foundation Trust, Manchester, Greater Manchester UK; 4 Department of Medicine, Lancashire Teaching Hospitals NHS Foundation Trust, Preston, England, UK; 5 Department of Neurosurgery, Manchester Centre for Clinical Neurosciences, Salford Royal Hospital, Manchester, UK; 6 High-Dimensional Neurology, Institute of Neurology, University College London, London, UK; 7 Department of Neurosurgery, National Hospital for Neurology and Neurosurgery, Victor Horsley, Queen Square, London, UK; 8 College of Letters and Sciences, University of California, Berkeley, CA 94720 USA; 9 Department of Geriatric Medicine, Salford Royal Hospital, Manchester, UK

**Keywords:** Chronic Subdural Haematoma, National Health Service, Nonagenarian, Octogenarian

## Abstract

**Background:**

The incidence of chronic subdural haematoma (CSDH) is rising in the elderly, yet there is limited research focusing on patients aged > 80. In the United Kingdom, the decision to transfer for neurosurgical intervention is dependent upon the acceptance of a CSDH management referral by a tertiary centre.

**Aims:**

This study thus aims to identify key predictors of referral acceptance in octogenarian and nonagenarian age groups, in comparison to the adult population.

**Methods:**

A multi-centre retrospective case series analysis from January 2015 to May 2020 was conducted. Patients were grouped as < 80 (‘adult’) or > 80 (‘ultra-geriatric’), with the latter further subgrouped into 80–89 (‘octogenarian’), and ≥ 90 (‘nonagenarian’). Multivariable logistic regression assessed age, headache, dementia, motor weakness, midline shift, CSDH size, and premorbid quality of life (QoL) to determine predictors of referral acceptance.

**Results:**

With a total of 3002 patient referrals, referral acceptance was significantly higher in adults compared to the ultra-geriatric group (38.1%vs22.4%, p < 0.001) and in octogenarians versus nonagenarians (27.7%vs10.5%, p = 0.036). CSDH size and premorbid QoL were consistent independent predictors across all age groups. In adults, all seven variables were significant predictors. For the ultra-geriatric group, all but headache and dementia were significant. Among octogenarians, motor weakness, midline shift, CSDH size, and premorbid QoL predicted acceptance, while in nonagenarians, only age, CSDH size, and QoL were significant.

**Conclusions:**

We demonstrate the importance of CSDH size and premorbid QoL as independent predictor variables for neurosurgical referral acceptance in all analysed age groups, particularly in the octogenarian and nonagenarian patient population, whilst other variables had variable significance depending upon age group.

## Introduction

Chronic subdural haematoma (CSDH) is one of the most common neurosurgical conditions affecting older patients [1]. The incidence of the condition has increased in recent decades and can be attributed to the ageing population and increasing use of anti-coagulant medication [2]. There is little evidence on the optimal management of these patients, however, surgical intervention is indicated in patients who deteriorate or do not improve [3]. In the United Kingdom (UK), neurosurgical care is centralized in tertiary centres and therefore CSDH patients often present to outlying emergency departments before they are referred to on-call neurosurgical services. The decision to accept a referral is complex and relies on both the patient’s baseline characteristics and the physician’s clinical judgment. This decision is particularly complex in older patients owing to their varying suitability for surgery. In 2001, the National Service Framework (NSF) reported on the complexity of caring for older adults in the hospital setting, highlighting the burden of polymorbidity and the need for early specialist intervention [4]. There is often debate regarding these patients’ suitability for surgery versus conservative management. These decisions are nuanced and may be challenging for junior trainees on call, who are often those responsible for triaging and operating on these patients [5]. The primary reason for these issues stem from the lack of literature surrounding the management of patients with CSDH, especially from a geriatric perspective [6].

Previous research has identified clinical and radiological variables that influence the outcome of CSDH referrals, including patient age, the presence of headache, dementia, motor weakness, midline shift, the size of the haematoma, and the patient’s pre-morbid quality of life (QoL) [6]. Surgical intervention remains the mainstay of treatment, with conservative management preferred in patients with small haematomas and in those with a lower pre-morbid QoL, who may not be suitable for surgery [7, 8]. Despite CSDH being a disease of older adults with a median age of 79 years, there are relatively few studies investigating the condition in an ‘ultra-geriatric’ population (> 80 years). We therefore aimed to complete a multicentre study to identify the important variables in predicting CSDH referral acceptance in octogenarian and nonagenarian patients and determine whether these variables were different to those below eighty years of age**.**

## Material and methods

### Guidelines

The study utilized the Strengthening the Reporting of Observational Studies in Epidemiology (STROBE) framework [9].

### Data source and feature selection

This retrospective multicentre study collected data from all CSDH patients referred to two high-volume tertiary neurosurgical units within the UK over a 5-year period from 2015 to 2020. The referral databases for each hospital were examined, and all CSDH referrals were exported in an anonymised manner for analysis. The patient cohort was divided into three groups: < 80-years-old (classified as ‘adult’), 80–89 years (octogenarian), and ≥ 90 years (nonagenarian). Patients aged 80–99 years were further classified as ‘ultra-geriatric’. No patients above 99 years (centenarians) were included in this analysis.

The study was vetted and approved by the hospital’s research and innovation team (Ref no: 22HIP11) and patient consent was deemed not necessary due to the blinded and anonymized nature of the study.

Data on 7 predictor variables at the point of index referral were extracted from the referral database: age of the patient (continuous), presence of headache (yes or no), dementia (yes or no), motor weakness (yes or no), midline shift (yes or no), the size of the CSDH (small, medium, or large), and the patient’s pre-morbid quality of life (QoL) (poor or reasonable). Radiological scans were used to demonstrate the presence of midline shift and haematoma size which was defined by the haematoma thickness: small (< 1 cm), medium (1–2 cm), and large (> 2 cm). The patient’s clinical frailty score (CFS) was not included as it was not uniformly included in referrals across the two centres. The patient’s pre-morbid QoL was used in place of CFS and is a composite measure of the patient’s mobility status, their requirement for full time care and the systemic burden of their co-morbidities which was scored according to their American Society of Anaesthesiologists (ASA) grade, as defined a priori by Biswas et al.[6] Poor pre-morbid QoL was defined as patients requiring full time care or those classified as an ASA Grade 4 [6]. The sex of the patient has been previously demonstrated to be a non-significant predictor of chronic subdural haematoma outcomes and thus was not included as a predictor variable in this study. The dichotomous outcome variable was the acceptance or rejection of a CSDH referral. Referral rejection meant that the patient was advised to have non-operative care in the local hospital and acceptance meant that the patient was to be transferred and admitted to the tertiary neurosurgical service for surgical intervention. Referral acceptance is strongly linked to surgical intent and in our clinical practice, accepted referrals are transferred specifically for operative consideration.

### Statistical analysis

IBM SPSS Statistics Software Version 28 for Mac OS and Windows OS, and the R coding language (R Foundation for Statistical Computing, Vienna, Austria) were utilised for all the statistical analysis. Continuous numerical data was expressed as mean ± standard deviation. Categorical data was expressed as frequency/percentage (%). All quantitative data was shown to have followed a Gaussian distribution. Multivariable stepwise feedforward binary logistic regression was performed to account for the influence of the predictor variables on the outcome class. Independent variables with univariable p < 0.10 were entered into the initial model, after which a forward (likelihood ratio) stepwise procedure was applied. Variables were added sequentially based on minimising the Akaike Information Criterion and retained only if they significantly improved model fit. A *p*-value of < 0.05 was considered significant.

## Results

### Baseline patient characteristics

**Fig. 1:**
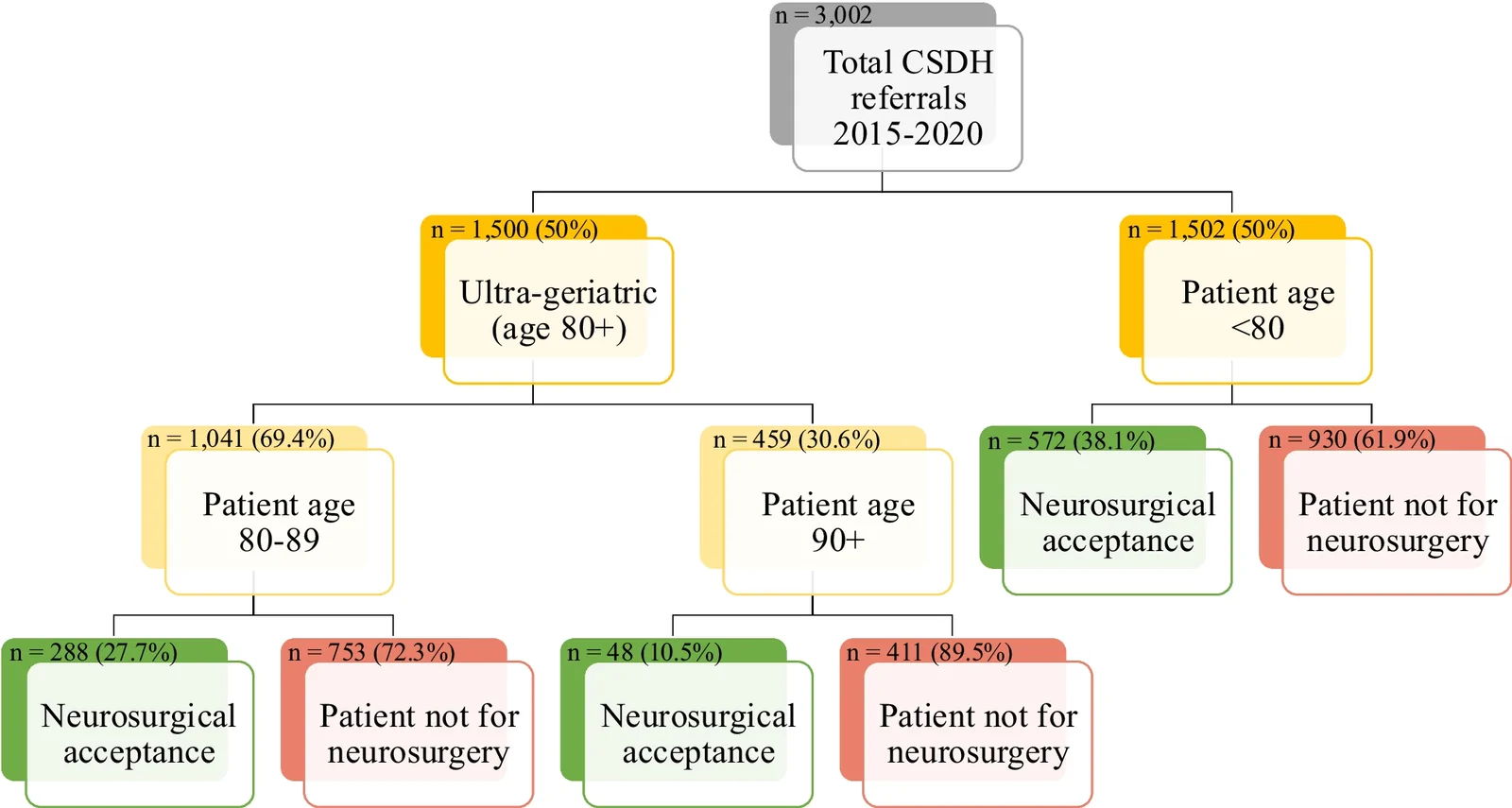
Patient cohort distribution

After exclusion of referrals with incomplete data (n = 211), a total of 3,002 patients were identified and included for analysis. Figure 1 demonstrates the cohort selection and referral outcomes. Demographics for this cohort have previously been reported, showing no significant differences between the patients characteristics of the two centres as well as the referral outcome rates [6, 10]. This demonstrates that the two centres had similar referral patterns and criteria for acceptance.

In the < 80-years-old cohort, 1,502 patients were identified, with a neurosurgical acceptance rate of 38.1% (572 patients). The mean age in this ‘adult’ cohort was 65.9 ± 12.6 years. The majority of patients had a reasonable pre-morbid QoL (88.7%) and did not have headaches (65.9%), dementia (92.8%) or motor weakness (74.8%). Radiologically, most patients presented with medium to large haematomas (53.1%), with midline shift present in 36.2% of patients (Table 1).

**Table 1:** Cohort demographics with comparative statistical analysis using t-tests for continuous variables (Mean (± Standard Deviation)) and chi-square tests for categorical variables (n (%)). *Represents *P* value < 0.05

	** < 80 s (n = 1502)**	**Ultra-geriatric (n = 1500)**	**Chi-squared value**	***P* value**	**Octogenarians (n = 1041)**	**Nonagenarians (n = 459)**	**Chi-squared value**	***P* value**
**Age**	65.9 ± 12.6	87.15 ± 4.9	NA	< 0.001*	84.5 ± 2.73	92 ± 2.46	NA	< 0.001*
**Headache**								
Yes	512 (34.1%)	170 (11.3%)	221.33	< 0.001*	133 (12.8%)	37 (8.1%)	7.05	0.030*
No	990 (65.9%)	1330 (88.7%)			908 (87.2%)	422 (91.9%)		
**Dementia**								
Yes	108 (7.2%)	364 (24.3%)	165.16	< 0.001*	231 (22.2%)	133 (29%)	7.98	0.196
No	1394 (92.8%)	1136 (75.7%)			810 (77.8%)	326 (71.1%)		
**Motor Weakness**								
Yes	378 (25.2%)	331 (22.1%)	3.99	0.046*	254 (24.4%)	77 (16.8%)	10.77	0.622
No	1124 (74.8%)	1169 (77.9%)			787 (75.6%)	382 (83.2%)		
**Midline Shift**								
Yes	543 (36.2%)	398 (26.5%)	32.27	< 0.001*	313 (30.1%)	85 (18.5%)	21.79	0.05
No	959 (63.8%)	1102 (73.5%)			728 (69.9%)	374 (81.5%)		
**Size of CSDH**								
Small	704 (46.9%)	831 (55.4%)	25.57	< 0.001*	536 (51.5%)	295 (64.3%)	29.60	0.568
Medium	277 (18.4%)	265 (17.7%)		< 0.001*	183 (17.6%)	82 (17.9%)		
Large	521 (34.7%)	404 (26.9%)		< 0.001*	322 (30.9%)	82 (17.9%)		
**Pre-morbid QoL**								
Poor	169 (11.3)	377 (25.1%)	97.19	< 0.001*	217 (20.8%)	260 (34.9%)	114.5	< 0.001*
Reasonable	1333 (88.7%)	1123 (74.9%)			824 (79.2%)	299 (65.1%)		
**Acceptance status**								
Accepted	572 (38.1%)	336 (22.4%)	87.49	< 0.001*	288 (27.7%)	48 (10.5%)	54.27	0.036*
Rejected	930 (61.9%)	1164 (77.6%)			753 (72.3%)	411 (89.5%)		

In the ‘ultra-geriatric’ population, 1,500 patients were identified, with 336 patients (22.4%) being accepted for specialist management. The mean age in this group was 87.15 ± 4.9 years. Baseline characteristics showed a similar trend to those of the adult cohort, with the majority of patients presenting without headache (88.7%), dementia (75.7%), motor weakness (77.9%), or midline shift (73.5%). Radiologically, most patients in this group had a small haematoma (55.4%). Chi-squared analysis comparing the distribution of baseline characteristics between the adult and the ultra-geriatric population revealed significant differences in the frequencies of all variables, with significantly fewer patients being accepted for neurosurgical intervention in the ultra-geriatric population (38.1% vs 22.4%, *p* = < *0.001*) (Table 1).

Subgroup analysis of the ultra-geriatric population identified 1,041 octogenarian and 459 nonagenarian patients. The mean age of these two groups were 84.5 ± 2.7 and 92 ± 2.5 years, respectively. Significantly more octogenarian patients were accepted for neurosurgical intervention (27.7% vs 10.5%, *p* = *0.036*). The majority of patients in the octogenarian cohort did not have headache (87.2%), dementia (77.8%), motor weakness (75.6%), or midline shift (69.9%). Most patients in this cohort presented with either a small (51.5%) or medium (17.6%) sized haematoma and a reasonable pre-morbid QoL (79.2%). In the nonagenarian cohort, most patients did not experience headache (91.9%), dementia (71.1%), or motor weakness (83.2%). Radiologically, the majority of nonagenarian patients presented with either a small (64.3%) or medium (17.9%) sized haematoma with no midline shift (81.5%). The only statistical difference in baseline characteristics was the frequency of headaches, which were more common in the octogenarian cohort (12.8% vs 8.1%, *p* = *0.030*) (Table 1).

### Predicting referral acceptance

In the adult cohort, the age of the patient (OR: 0.970, *p* = 0.002), headache (OR: 2.647, *p* = < 0.001), dementia (OR: 0.240, *p* = < 0.011), motor weakness (OR: 1.940, *p* = < 0.012), midline shift (OR: 5.847, *p* = < 0.001), CSDH size (OR: 19.906, *p* = < 0.001), and the pre-morbid QoL of the patient (OR: 0.015*, p* = < 0.001) were all statistically significant predictors of neurosurgical referral acceptance. With the exception of headache (OR: 0.753, *p* = 0.389) and dementia (OR: 0.621, *p* = 0.116), the same variables were also identified as significant predictors of acceptance in the ultra-geriatric population.

Subgroup analysis of the ultra-geriatric population identified motor weakness (OR: 2.810, *p* = < 0.001), midline shift (OR: 5.886, *p* = < 0.001), CSDH size (OR: 16.313, *p* = < 0.001), and pre-morbid QoL (OR: 0.024, *p* = < 0.001) as significant predictors of neurosurgical acceptance in octogenarian patients. In contrast, for the nonagenarian cohort, patient age (OR: 0.731, *p* = 0.020), CSDH size (OR: 47.832, *p* = < 0.001), and pre-morbid QoL (OR: 0.068, *p* = < 0.001) were the only significant predictors for neurosurgical intervention (Table 2).

**Table 2:** Logistic regression analysis for the ultra-geriatric (n = 1614), < 80 s (n = 1502), octogenarian (n = 1041), and nonagenarian (n = 459) cohorts. *Represents *P* value < 0.05. OR = Odds Ratio, Cl = Confidence Interval

Characteristics	OR	95% CI	P-value
** < 80 s cohort**			
Age	0.970	0.951—0.989	0.002*
Headache	2.647	1.649—4.249	< 0.001*
Dementia	0.240	0.080—0.722	0.011*
Motor Weakness	1.940	1.158—3.251	0.012*
Midline Shift	5.847	3.654—9.354	< 0.001*
CSDH size	19.906	13.603—29.129	< 0.001*
Pre-morbid QoL	0.015	0.006—0.042	< 0.001*
**‘Ultra-geriatric’ cohort**			
Age	0.494	0.296—0.825	0.007*
Headache	0.753	0.395—1.436	0.389
Dementia	0.621	0.343—1.125	0.116
Motor Weakness	2.755	1.661—4.567	< 0.001*
Midline Shift	4.503	2.775—7.039	< 0.001*
CSDH size	18.522	12.303—27.885	< 0.001*
Pre-morbid QoL	0.022	0.011—0.045	< 0.001*
**Octogenarian cohort**			
Age	0.960	0.868—1.063	0.436
Headache	0.935	0.442—1.979	0.860
Dementia	0.667	0.325—1.368	0.269
Motor Weakness	2.810	1.498—5.273	< 0.001*
Midline Shift	5.886	3.274—10.581	< 0.001*
CSDH size	16.313	10.293—25.855	< 0.001*
Pre-morbid QoL	0.024	0.010—0.059	< 0.001*
**Nonagenarian cohort**			
Age	0.731	0.562—0.951	0.020*
Headache	0.184	0.031—1.106	0.064
Dementia	0.478	0.146—1.568	0.223
Motor Weakness	2.296	0.789—6.685	0.127
Midline Shift	1.250	0.400—3.904	0.700
CSDH size	47.832	13.031—175.566	< 0.001*
Pre-morbid QoL	0.068	0.018—0.257	< 0.001*

## Discussion

To our knowledge this is the largest study to analyse the referral process for chronic subdural haematoma (CSDH) patients in an ‘ultra-geriatric’ population. Patients with increased age are frailer and suffer from multiple co-morbidities that place them at an increased risk of in-hospital mortality and post-operative complications compared to younger patients [11]. There are currently no established international guidelines regarding the clinical decision-making process for admitting geriatric patients with CSDH for surgical management. Current research in the literature has focussed on surgical outcomes and predicting haematoma recurrence rates. Our study highlights associations between the significant predictors of acceptance for neurosurgical intervention in ultra-geriatric patients and offers insight into factors that may influence clinicians managing this heterogenous population. However, as this study was conducted in the UK healthcare context, the findings may have limited generalizability to international settings.

### Predictors for CSDH referral acceptance

Across all studied ultra-geriatric patients, with the exception of the nonagenarian cohort, the most significant variables in association with CSDH referral acceptance were the size of the haematoma and presence of midline shift. This observation is in keeping with other studies which have reported associations larger haematoma size and the presence of midline shift with worse patient outcomes, often prompting consideration of neurosurgical intervention [5–8, 10, 12–16]. Additionally, haematoma size has been associated with symptomatology, with smaller haematomas being more likely to be treated conservatively due to their low neurological morbidity [3, 16]. Similarly, our data reflects the findings of Brenan et al. which highlight the primary reason for referral rejection as haematoma size that is insufficient to explain patient symptomatology [17]. There are several reasons why the presence of midline shift may not be a significant predictor of acceptance in nonagenarian patients. Khorasanizadeh et al., have described the inverse effect of age, accounting for haematoma volume, on midline shift. They report significantly lower levels of midline shift in patients older than 75 years, possibly as a result of higher parenchymal compliance and a larger subdural space because of increased cerebral atrophy as contributory factors [18]. This is in keeping with our observations, where only 18.5% of nonagenarian patients had midline shift compared to 36.2% and 30.1% for the adult and octogenarian cohorts respectively.

Moreover, we identified increasing age as a negative predictor for referral acceptance with our rejected cohort having a significantly greater number of patients older than 80 years. This trend was most evident within the ultra-geriatric population, where only 10.5% of nonagenarian patients were accepted for neurosurgical intervention, compared to 38.1% and 27.7% in the adult and octogenarian cohorts respectively. It is well documented that older patients over the age of 75 years undergoing surgical intervention for CSDH experience increased mortality and morbidity when compared to younger patients [19–21]. Toi et al. reported frequencies of poor outcomes at discharge of 37.4% and 56.8% for patients in their 80’s and 90’s, respectively, when compared to rates of 11.7–29.4% in those below 80 years of age [22]. Brennan *et a*l. observed an association of increased age with worse functional outcomes post-operatively but highlighted functional improvement in select patients [17]. These findings are likely related to the burden of multiple co-morbidities, worse pre-morbid QoL, and reduced physiological reserve associated with increased age [23–25]. Interestingly, for octogenarian patients, age was not a significant factor in predicting referral acceptance. Recent studies suggest that, despite experiencing more early post-operative complications, most of these patients have favourable functional outcomes and highlight that age should not be a negative determinant of neurosurgical intervention [24, 26, 27]. As discussed previously, a greater proportion of ultra-geriatric patients had small/medium sized haematomas without midline shift. It may be possible that the age-related increase in the size of the subdural space may allow for greater expansion of the haematoma without increasing intracranial pressure. Therefore, neurosurgical intervention in this cohort may only be beneficial once the haematoma has reached a critical size that causes patient deterioration. This may also explain the non-significance and lower frequency of patient symptoms (motor weakness and headache) in the nonagenarian population.

In our study, dementia was a significant negative predictor for neurosurgical intervention in the adult cohort. In the literature, the presence of dementia is associated with increased length of stay and discharge to supported living, however, the effects of dementia on post-operative mortality and morbidity are inconsistently reported [28–31]. Despite the presence of dementia, other variables such as pre-morbid QoL and CSDH size are far more important in their associated with the need for intervention in this study. For example, even if there was mild dementia but the pre-morbid QoL was reasonable, intervention was more likely. It is also possible that the observed association with rejection is due to the collinearity between dementia and increasing age, which was also a negative predictor of intervention.

In our study, pre-morbid QoL was a composite measure of the patient’s mobility status, their requirement for full time care and the systemic burden of their co-morbidities on their health – all of which are indirect markers of frailty. We observed a significant association between reasonable pre-morbid QoL and referral acceptance across all cohorts which is in keeping with the literature which highlights the importance of patient comorbidities and frailty status on their suitability for surgery [23–25, 32–35]. Comorbid patients are more likely to suffer from adverse outcomes and increased morbidity post operatively. De Bonis et al*.* report significant associations with high Charlson comorbidity index scores with worse mortality and complication rates at 6 months post-op [36]. Moreover, there is debate as to what extent CSDH is a benign condition. Recently, it has been termed a ‘sentinel event’ and has been associated with 1 year mortality rates of ~ 30%, cautioning intervention in those with high disease burden as is common in ultra-geriatric patients [37]. These findings highlight the need for a flexible approach in assessing ultra-geriatric patients with CSDH accounting for their pre-morbid functioning. More research is needed to assess the impact of pre-morbid QoL and burden of multiple co-morbidities on mortality and morbidity in ultra-geriatric patients.

### Limitations

Despite our findings, our study suffers from some limitations. Firstly, our results may be biased. The referral decision made by the consultant neurosurgeon was assumed to be correct with no further independent verification of this. It is however important to note that this reflects standard practice across neurosurgical emergency care, and we have tried to account for such variation in individual decision making by including referrals assessed by more than 40 neurosurgeons across the two centres.

No follow-up data was collected as part of this study and hence it is not clear exactly what percentage of the patients who were accepted for transfer eventually underwent surgery. Our clinical experience suggests this to be the case for only a minority of patients and referral acceptance usually serves as a proxy for operative intervention. Furthermore, we acknowledge the potential inter-centre variabilities that may exist and re-iterate the importance of external validation of our results. Thus, these results should be interpreted as associative and further external validation and non-stepwise modelling approaches are required.

Moreover, we only analysed variables that were significant on previous analysis [6, 10]. It is therefore possible that factors such as ethnicity, sex, specific co-morbidities (cardiovascular disease, diabetes, respiratory disease, etc.), previous history of CSDH, other neurological symptoms, and type of anticoagulation are significant variables that could influence the outcome of referrals. However, it is important to note that previous work on factors such as anticoagulant use, show that it does not typically influence whether a patient is accepted or rejected, largely because the management approach in these cases is almost always to suspend or reverse anticoagulation regardless. Furthermore, due to differences in referral systems between the two centres, clinical frailty status could not be included as an individual variable and to maintain a straightforward model suitable for routine clinical use, we elected to use ASA grade as a global indicator of comorbidity burden, operative risk, and pre-morbid functional reserve.

### Future directions

Whilst this study has shown the nuances in decision making when assessing older patients with CSDH, more research is required to assess how pre-operative clinical variables correlate with post-operative morbidity and mortality. Most notably, pre-morbid quality of life was a significant predictor of acceptance in this study. It is therefore imperative that future work looking at post-operative outcomes in this vulnerable population focus on post-operative QoL, frailty, and ability to complete activities of daily living. Our group is currently in the process of obtaining such data and aim to perform more in-depth analysis looking at both pre-operative and post-operative clinical variables to determine whether patients are suitable candidates for neurosurgical intervention and perform longitudinal analysis of these patients to determine post-operative outcomes.

## Conclusion

This is the largest study to investigate the variables predicting referral acceptance for ultra-geriatric patients with CSDH. We highlight the importance of pre-morbid QoL and haematoma size in selecting patients for neurosurgical intervention in this cohort, rather than using age as a sole determining factor. More research is required to determine how the investigated variables impact post-operative mortality and morbidity. In the long-term, this data can help influence the development of clinical decision-making guidelines, to aid the holistic management of an increasingly ageing, multi-morbid population, at ever-increasing risk of CSDH.

## Data Availability

Data is available upon reasonable request to the corresponding authors.

## References

[1] Edlmann E, Giorgi-Coll S, Whitfield PC, Carpenter KLH, Hutchinson PJ (2017) Pathophysiology of chronic subdural haematoma: inflammation, angiogenesis and implications for pharmacotherapy. J Neuroinflammation 14:108. 10.1186/s12974-017-0881-y PMC5450087 28558815

[2] Rust T, Kiemer N, Erasmus A (2006) Chronic subdural haematomas and anticoagulation or anti-thrombotic therapy. J Clin Neurosci 13:823–827. 10.1016/j.jocn.2004.12.013 16997707

[3] Feghali J, Yang W, Huang J (2020) Updates in chronic subdural haematoma: epidemiology, etiology, pathogenesis, treatment, and outcome. World Neurosurg 141:339–345. 10.1016/j.wneu.2020.06.140 32593768

[4] Ford P, Wild D, Dewing J (2001) National service framework a GNP response. Nurs Older People 13:30–31. 10.7748/nop.13.6.30.s16 27707080

[5] Abouzari M, Rashidi A, Rezaii J, Esfandiari K, Asadollahi M, Aleali H, Abdollahzadeh M (2007) The role of postoperative patient posture in the recurrence of traumatic chronic subdural haematoma after burr-hole surgery. Neurosurgery 61:794–797. 10.1227/01.NEU.0000298908.94129.67 17986941

[6] Biswas S, MacArthur J, Sarkar V and others (2022) Development and validation of the Chronic subdural Haematoma Referral oUtcome prediction using Statistics (CHORUS) score: A retrospective study at a national tertiary centre. World Neurosurg 170:e724-36. 10.1016/j.wneu.2022.11.097 36442777

[7] Posti JP, Luoto TM, Sipilä JOT and others (2022) Prognosis of patients with operated chronic subdural hematoma. Sci Rep 12:7020. 10.1038/s41598-022-10992-5 PMC9054845 35488040

[8] Shimizu K, Sadatomo T, Hara T, Onishi S, Yuki K, Kurisu K (2018) Importance of frailty evaluation in the prediction of the prognosis of patients with chronic subdural haematoma. Geriatr Gerontol Int 18:1173–1176. 10.1111/ggi.13436 29770549

[9] von Elm E, Altman DG, Egger M, Pocock SJ, Gøtzsche PC, Vandenbroucke JP (2007) The strengthening the reporting of observational studies in epidemiology (STROBE) statement: guidelines for reporting observational studies. Lancet 370:1453–1457. 10.1016/S0140-6736(07)61602-X 18064739

[10] Biswas S, MacArthur JI, Pandit A, McMenemy L, Sarkar V, Thompson H, Saleemi MS, Chintzewen J, Almansoor ZR, Chai XT, Hardman E, Torrie C, Holt M, Hanna T, Sobieraj A, Toma A, George KJ (2023) Predicting neurosurgical referral outcomes in patients with chronic subdural haematomas using machine learning algorithms - A multi-center feasibility study. Surg Neurol Int 14:22. 10.25259/SNI_1086_2022 PMC9899452 36751456

[11] Clegg A, Young J, Iliffe S, Rikkert MO, Rockwood K (2013) Frailty in elderly people. Lancet 381:752–762. 10.1016/S0140-6736(12)62167-9 PMC4098658 23395245

[12] Bechstein M, McDonough R, Fiehler J, Zanolini U, Rai H, Siddiqui A, Shotar E, Rouchaud A, Goyal M, Gellissen S (2022) Radiological evaluation criteria for chronic subdural hematomas: review of the literature. Clin Neuroradiol 32:923–929. 10.1007/s00062-022-01138-1 PMC9744701 35157088

[13] Kim HC, Ko JH, Yoo DS, Lee S-K (2016) Spontaneous resolution of chronic subdural haematoma: close observation as a treatment strategy. J Korean Neurosurg Soc 59:628–636. 10.3340/jkns.2016.59.6.628 PMC5106364 27847578

[14] Leroy H-A, Aboukaïs R, Reyns N, Bourgeois P, Labreuche J, Duhamel A, Lejeune JP (2015) Predictors of functional outcomes and recurrence of chronic subdural hematomas. J Clin Neurosci 22:1895–1900. 10.1016/j.jocn.2015.03.064 26260114

[15] Miah IP, Tank Y, Rosendaal FR, Peul WC, Dammers R, Lingsma HF, den Hertog HM, Jellema K, van der Gaag NA (2021) Radiological prognostic factors of chronic subdural haematoma recurrence: a systematic review and meta-analysis. Neuroradiology 63:27–40. 10.1007/s00234-020-02558-x PMC7803717 33094383

[16] Yamamoto H, Hirashima Y, Hamada H, Hayashi N, Origasa H, Endo S (2003) Independent predictors of recurrence of chronic subdural haematoma: results of multivariate analysis performed using a logistic regression model. J Neurosurg 98:1217–1221. 10.3171/jns.2003.98.6.1217 12816267

[17] Brennan PM, Kolias AG, Joannides AJ, Shapey J, Marcus HJ, Gregson BA, Grover PJ, Hutchinson PJ, Coulter IC (2017) The management and outcome for patients with chronic subdural haematoma: a prospective, multicenter, observational cohort study in the United Kingdom. J Neurosurg 127:732–739. 10.3171/2016.8.JNS16134 28306417

[18] Khorasanizadeh M, Chang Y-M, Enriquez-Marulanda A, Khorasanizadeh MH, Mizuhashi S, Salem MM, Gomez-Paz S, Siddiq F, Kan P, Moore J, Ogilvy CS, Thomas AJ (2022) Morphological changes in chronic subdural haematomas following upfront middle meningeal artery embolization: sequence, timing, and association with outcomes. J Neurosurg 137:235–248. 10.3171/2021.8.JNS211691 34826814

[19] Borger V, Vatter H, Oszvald Á, Marquardt G, Seifert V, Güresir E (2012) Chronic subdural haematoma in elderly patients: a retrospective analysis of 322 patients between the ages of 65-94 years. Acta Neurochir (Wien) 154:1549–1554. 10.1007/s00701-012-1434-x 22772400

[20] Karibe H, Kameyama M, Kawase M and others (2011) Epidemiology of chronic subdural haematomas. No Shinkei Geka 39:1149–1153 22128269

[21] Ro HW, Park SK, Jang DK, Yoon WS, Jang KS, Han YM (2016) Preoperative predictive factors for surgical and functional outcomes in chronic subdural hematoma. Acta Neurochir (Wien) 158:135–139. 10.1007/s00701-015-2625-z 26602235

[22] Toi H, Kinoshita K, Hirai S, Takai H, Hara K, Matsushita N, Matsubara S, Otani M, Muramatsu K, Matsuda S, Fushimi K, Uno M (2018) Present epidemiology of chronic subdural haematoma in Japan: analysis of 63,358 cases recorded in a national administrative database. J Neurosurg 128:222–228. 10.3171/2016.9.JNS16623 28156246

[23] Aceto P, Antonelli Incalzi R, Bettelli G, Carron M, Chiumiento F, Corcione A, Crucitti A, Maggi S, Montorsi M, Pace MC, Petrini F, Tommasino C, Trabucchi M, Volpato S (2020) Perioperative management of elderly patients (PriME): recommendations from an Italian intersociety consensus. Aging Clin Exp Res 32:1647–1673. 10.1007/s40520-020-01624-x PMC7508736 32651902

[24] Lin H-S, Watts JN, Peel NM, Hubbard RE (2016) Frailty and post-operative outcomes in older surgical patients: a systematic review. BMC Geriatr 16:157. 10.1186/s12877-016-0329-8 PMC5007853 27580947

[25] Pearce L, Bunni J, McCarthy K, Hewitt J (2016) Surgery in the older person: training needs for the provision of multidisciplinary care. Ann R Coll Surg Engl 98:367–370. 10.1308/rcsann.2016.0180 PMC5209983 27269239

[26] Christopher E, Poon MTC, Glancz LJ, Hutchinson PJ, Kolias AG, Brennan PM (2019) Outcomes following surgery in subgroups of comatose and very elderly patients with chronic subdural haematoma. Neurosurg Rev 42:427–431. 10.1007/s10143-018-0979-4 PMC6502770 29679178

[27] Watanabe S, Kato N, Sato M, Aiyama H, Fujiwara Y, Goto M, Yamazaki T, Yasuda S, Ishikawa E, Matsumura A (2019) Treatment outcomes of burr-hole surgery for chronic subdural haematoma in the elderly living beyond life expectancy: a study comparing cure, recurrence, and complications in patients aged ≥80 years versus ≤79 years. World Neurosurg 132:e812–e819. 10.1016/j.wneu.2019.08.003 31404689

[28] Bekelis K, Missios S, Shu J, MacKenzie TA, Mayerson B (2018) Surgical outcomes for patients diagnosed with dementia: a coarsened exact matching study. J Clin Neurosci 53:160–164. 10.1016/j.jocn.2018.04.052 PMC6091887 29739725

[29] Clark CJ, Adler R, Xiang L, Shah SK, Cooper Z, Kim DH, Lin KJ, Hsu J, Lipsitz S, Weissman JS (2023) Outcomes for patients with dementia undergoing emergency and elective colorectal surgery: a large multi-institutional comparative cohort study. Am J Surg 226:108–114. 10.1016/j.amjsurg.2023.03.012 PMC10330079 37031040

[30] Kassahun WT (2018) The effects of pre-existing dementia on surgical outcomes in emergent and nonemergent general surgical procedures: assessing differences in surgical risk with dementia. BMC Geriatr 18:153. 10.1186/s12877-018-0844-x PMC6029045 29970028

[31] Masutani R, Pawar A, Lee H, Weissman JS, Kim DH (2020) Outcomes of common major surgical procedures in older adults with and without dementia. JAMA Netw Open 3:e2010395. 10.1001/jamanetworkopen.2020.10395 PMC7361650 32662842

[32] Ayyash R, Knight J, Kothmann E, Eid M, Ayyash K, Colling K, Yates D, Mill A, Danjoux G (2022) Utility and reliability of the Clinical Frailty Scale in patients scheduled for major vascular surgery: a prospective, observational, multicentre observer-blinded study. Perioper Med 11:6. 10.1186/s13741-022-00240-9 PMC8802497 35101117

[33] Gu C, Lu A, Lei C, Wu Q, Zhang X, Wei M, Wang Z (2022) Frailty index is useful for predicting postoperative morbidity in older patients undergoing gastrointestinal surgery: a prospective cohort study. BMC Surg 22:57. 10.1186/s12893-022-01471-9 PMC8851713 35172806

[34] Montgomery C, Stelfox H, Norris C, Rolfson D, Meyer S, Zibdawi M, Bagshaw S (2021) Association between preoperative frailty and outcomes among adults undergoing cardiac surgery: a prospective cohort study. CMAJ Open 9:E777–E787. 10.9778/cmajo.20200034 PMC8313095 34285057

[35] Partridge JSL, Harari D, Dhesi JK (2012) Frailty in the older surgical patient: a review. Age Ageing 41:142–147. 10.1093/ageing/afr182 22345294

[36] De Bonis P, Olei S, Mongardi L, Cavallo MA, Santantonio M, Trevisi G, Anile C, Mangiola A (2018) Chronic subdural hematoma in patients aged 80 years and older: a two-centre study. Clin Neurol Neurosurg 170:88–92. 10.1016/j.clineuro.2018.05.002 29753169

[37] Dumont TM, Rughani AI, Goeckes T, Tranmer BI (2013) Chronic subdural haematoma: a sentinel health event. World Neurosurg 80:889–892. 10.1016/j.wneu.2012.06.026 22722034

